# Simultaneous Determination of Multiple Mycotoxins in Swine, Poultry and Dairy Feeds Using Ultra High Performance Liquid Chromatography-Tandem Mass Spectrometry

**DOI:** 10.3390/toxins12040253

**Published:** 2020-04-13

**Authors:** Kraiwut Nualkaw, Saranya Poapolathep, Zhaowei Zhang, Qi Zhang, Mario Giorgi, Peiwu Li, Antonio Francesco Logrieco, Amnart Poapolathep

**Affiliations:** 1 Department of Pharmacology, Faculty of Veterinary Medicine, Kasetsart University, Bangkok 10900, Thailand; kraiwut.n@dld.go.th (K.N.); fvetsys@ku.ac.th (S.P.); 2 Oil Crops Research Institute of the Chinese Academy of Agricultural Sciences, Wuhan 430062, China; zhaowei_zhang@126.com (Z.Z.); zhangqi01@caas.cn (Q.Z.); peiwuli@oilcrops.cn (P.L.); 3 Department of Veterinary Science, University of Pisa, 56124 Pisa, Italy; mario.giorgi@unipi.it; 4 Institute of Sciences of Food Production, National Research Council, 70126 Bari, Italy; antonio.logrieco@ispa.cnr.it

**Keywords:** multi-mycotoxins, QuEChERS, animal feeds, UHPLC-MS/MS

## Abstract

A reliable, sensitive and accurate multiple mycotoxin method was developed for the simultaneous determination of 17 mycotoxins in swine, poultry and dairy feeds using stable isotope dilution (^13^C-ISTD) and (ultra)-high performance liquid chromatography-tandem mass spectrometry (HPLC-MS/MS). A simple QuEChERS-based method (quick, easy, cheap, effective, rugged and safe) was developed consisting of soaking with a solution of 1% formic acid followed by extraction with acetonitrile, clean-up with C18 sorbent and finally adding ^13^C-ISTD before the UHPLC-MS/MS analysis. The chromatographic condition was optimized for separation and detection of the 17 mycotoxins using gradient elution. The method’s performance complied with the SANTE/11813/2017 standard and had mean recovery accuracies in the range 70%–120% and precision testing of % relative standard deviation (RSD) ≤ 20%. The limit of detection and limit of quantification values ranged from 0.25 to 40.0 ng/g and 0.5 to 100.0 ng/g, respectively. Finally, the method was applied to analyze feed samples, with the results showing that fumonisins, zearalenone, aflatoxin B1 and deoxynivalenol were the most prevalent mycotoxins contaminating the feed samples.

## 1. Introduction

Mycotoxins are toxic secondary metabolites produced by filamentous fungi, especially by the *Aspergillus*, *Fusarium* and *Penicillium* genera that contaminate agricultural commodities and animal feeds [1]. Nowadays, more than 300 mycotoxins with various types of toxicity (including immunosuppressive, hepatoxic, mutagenic, carcinogenic and estrogenic effects) in mammals have been identified in agricultural products and result in substantial adverse economic impacts [2,3]. The Food and Agriculture Organization of the United Nations estimated that approximately 25% of the cereals produced in the world are contaminated by mycotoxins [4]. Recently, it is estimated that about 60%–80% of crops around the world are contaminated with mycotoxins at detectable levels [5]. Animal feed products have been regulated only for aflatoxin B1 (AFB1) by legislation through the European Commission Decision 2002/32/EC [6] and limits have been set for deoxynivalenol (DON), ochratoxin A (OTA), zearalenone (ZEA), T-2 toxin (T-2), HT-2 toxin (HT-2), fumonisin B1 (FB1) and fumonisin B2 (FB2) [7].

To date, the reliable detection and quantification of different types of mycotoxins in complicated food and feed matrices requires well-performed analytical procedures including suitable sample preparation methods and highly sensitive instrument. Several studies have reported the development of the method for the analysis of multiclass mycotoxins in feeds [8,9,10,11,12,13,14,15]. Currently, the best analytical technique for sensitive and quantification of multiclass mycotoxins in animal feed is (ultra-)high performance liquid chromatography coupled with tandem mass spectrometry, (U-)HPLC-MS/MS. The reversed phase LC-MS/MS system with the stationary phase as a C18 separation column has become the most popular for the multi mycotoxin analysis of animal feed matrices [1,8,16,17]. However, the mobile phase composition for LC-MS/MS analysis is different. For example, Malachova et al. (2014) [18] used 5 mM ammonium acetate (AmAc) with 1% (v/v) of acetic acid (AA) in both aqueous and methanol (MeOH) solutions. Lacina et al. (2012) and Dzuman et al. (2014) [8,19] used the mobile phases composition depend on the polarity of ESI (+) and ESI (−) analysis. 

The vital step and the main challenge in using the UHPLC-MS//MS method is the optimization of the sample preparation procedure. Solid liquid extraction (SLE) with acidified acetonitrile without a clean-up step has been applied to extract multiclass mycotoxins in various feed samples [1,16]. In the clean-up step, several researchers used either solid phase extraction (SPE) with a cartridge (Mycosep, C_18_) or liquid–liquid partitioning with hexane to enable defatting of the extract [11,12]. The sample preparation method, namely QuEChERS (quick, easy, cheap, effective, rugged and safe), consists of two steps: i) a solid–liquid extraction/partitioning with a salting-out effect and ii) a dispersive solid-phase extraction (d-SPE) for sample cleanup purposes [20]. This method was first introduced at the European Pesticide Residue Workshop 2002 in Rome [21]. The original procedure was developed to analyze multiple pesticide residues from fruit and vegetables; it has been applied to multiclass mycotoxins analysis in animal feeds by several researchers [8,17]. Sodium acetate buffer was used for analytes isolation with phase partitioning after the extraction using acidified acetonitrile with formic acid for the analysis of multiclass mycotoxins, veterinary drugs and pesticides in different complex matrices, including maize, feeds, meats, eggs and honey. Dzuman et al. (2015) [22] used a 2% solution of formic acid for soaking, then extracted using acetonitrile, C_18_ sorbent and magnesium sulfate (MgSO_4_) to remove any interference from the matrices co-extracted. This method was also validated in various samples, including calf feed, maize silage and wheat.

However, interference from the matrices can induce signal suppression and enhancement of the analyte during the ionization process, leading to incorrect results [23]. To compensate for the matrix effect and to increase the method’s accuracy, some methods developed for analysis of mycotoxins in animal feed, baby foods [13,24,25,26], maize [27], beer [28] and wine [29] have used an isotopically labeled internal standard. For the combination of QuEChERS and immunoaffinity column clean-up with isotopically labeled internal standard was performed to analyze mycotoxin in foods [30].

The purpose of the present study was to develop and validate a rapid and effective multiple analytical method for mycotoxins based on QuEChERS procedures by applying stable isotope dilution using HPLC-MS/MS for the simultaneous determination in complete feed including swine, poultry and dairy feeds of 17 mycotoxins: AFB1, aflatoxin B2 (AFB2), aflatoxin G1 (AFG1), aflatoxin G2 (AFG2), FB1, FB2, DON, nivalenol (NIV), 3-acetyldeoxynivalenol (3-AcDON), 15-acetyldeoxynivalenol (15-AcDON), fusarenon-X (FusX), T-2, HT-2, diacetoxyscirpenol (DAS), neosolaniol (NEO), OTA and ZEA.

## 2. Results and Discussion

### 2.1. LC-MS/MS Analysis

Amounts of 5 mM ammonium formate (AmF) and 0.2% formic acid (FA) in both Milli-Q water and methanol were used in ESI (+), whereas 5 mM AmAc in Milli-Q water and pure MeOH were used for ESI (−). In the first stage of our study, the two above-mentioned mobile phases were tested for analytes detection in ESI (+) and ESI (−).

For ESI (+), the 12 analytes including the isotopical mycotoxins were detected in ESI (+) especially for AFs that were at levels even more than 10 times that using an AmF solution with FA compared to the use of AmAc with 1% AA. The chromatogram for the FBs had good peak shapes and the highest sensitivity using AmF with 0.1% FA. 

**Figure 1:**
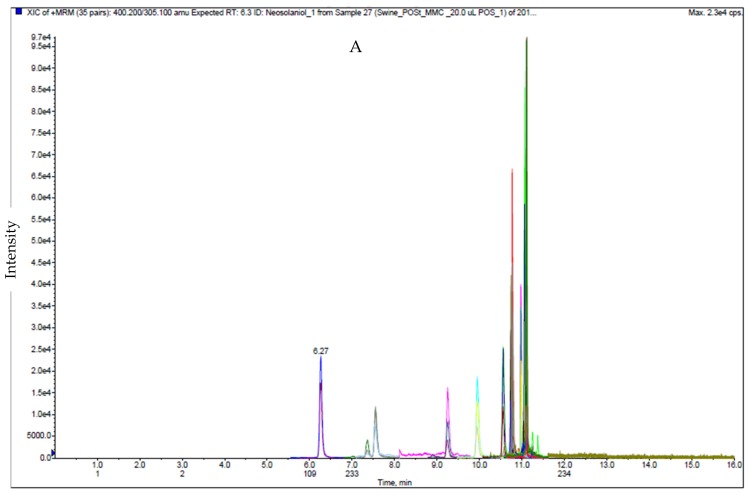

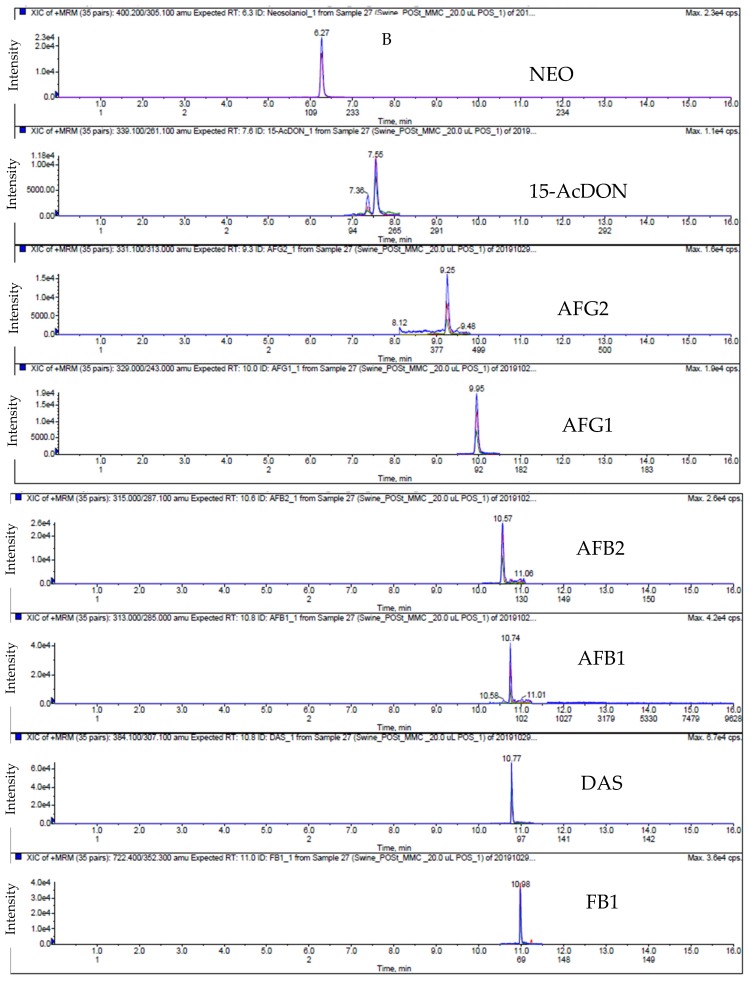

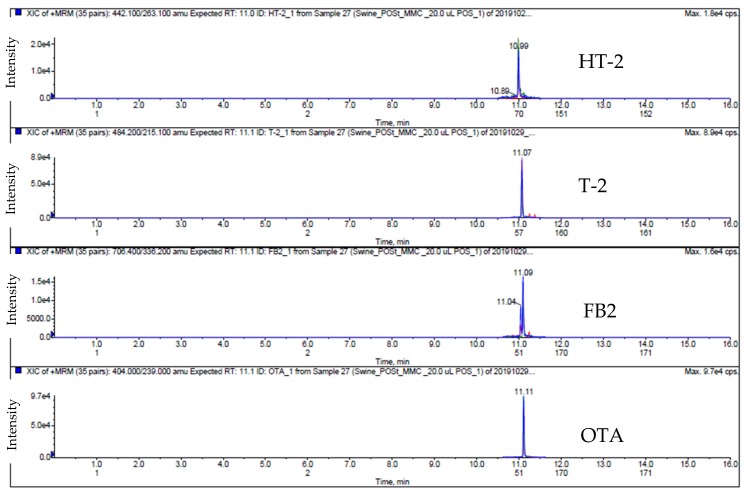
Ion chromatogram of matrix match calibration standard of swine feed samples for 12 native mycotoxin and 11 isotopically mycotoxin under optimized condition in ESI positive; 1.0 ng/mL for AFB1, AFB2, AFG1 and AFG2, with 8.0 ng/mL for T-2, HT-2, DAS, NEO, and OTA, 40.0 ng/mL for 15-AcDON, 30.0 ng/mL for FB1 and 9.0 ng/mL for FB2. **A**: Total Ion Chromatogram (TIC) and **B**: Extract Ion Chromatogram (EIC)

**Figure 2:**
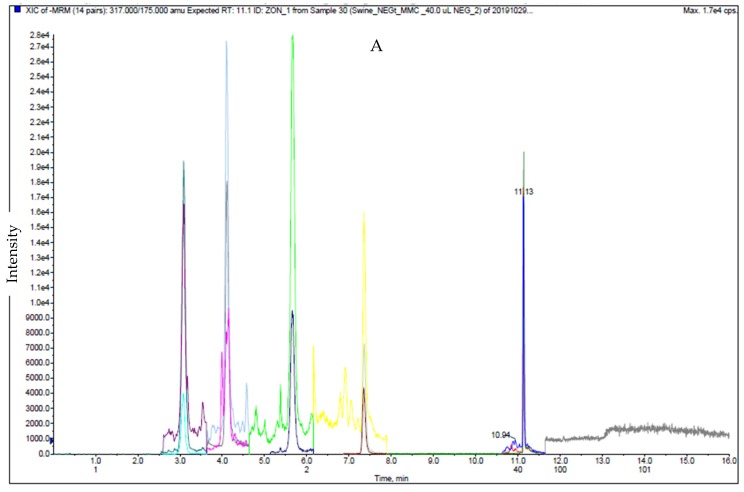

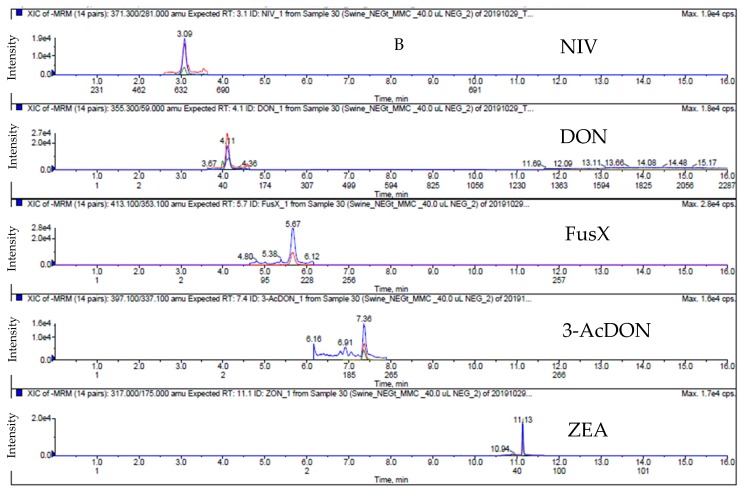
Ion chromatogram of matrix match calibration standard for swine feed samples for 5 native mycotoxins and 4 isotopical mycotoxins under optimized conditions in ESI negative (−); 1.0 ng/mL for ZEA, 40.0 ng/mL for DON, 3-AcDON, NIV and FuSX. **A**: Total Ion Chromatogram (TIC) and **B**: Extract Ion Chromatogram (EIC)

For ESI (−), the type B trichothecenes (except 15-AcDON and ZEA) were detected and had the highest sensitivity using the acetate buffer solution with acetic acid for the mobile phase [18,31]. Amounts of 5 mM AmF and 0.1% FA in both Milli-Q water and methanol solutions were used in ESI (+), whereas 5 mM AmF and 0.1% AA in both Milli-Q water and methanol solutions were used for ESI (−). Keeping the same ionic strength over the gradient was essential to improve the peak shape and retention time reproducibility. The developed method showed the better separation for acetylated deoxynivalenol (3-AcDON and 15AcDON) than those in different ionization mode [16]. The extract ion chromatograms (EIC) of the 17 mycotoxins including 15 isotopically internal standards in ESI (+) and ESI (−) are illustrated in Figure 1 and Figure 2, respectively.

### 2.2. QuEChERS-Based Procedure

**Figure 3:**
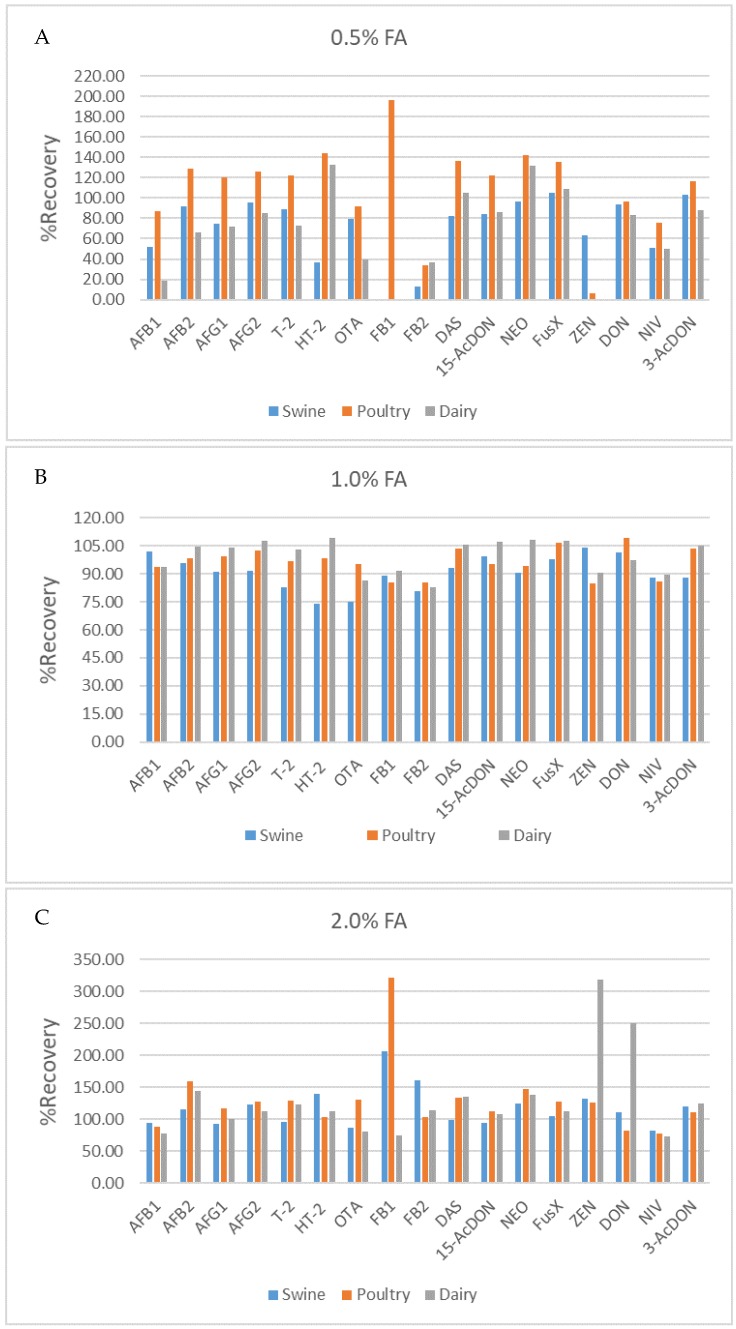
%Recovery for mycotoxin comparison on %formic acid (FA) on soaking experiment; **A**: 0.5% FA. **B**: 1.0% FA and **C**: 2.0% FA.

**Figure 4:**
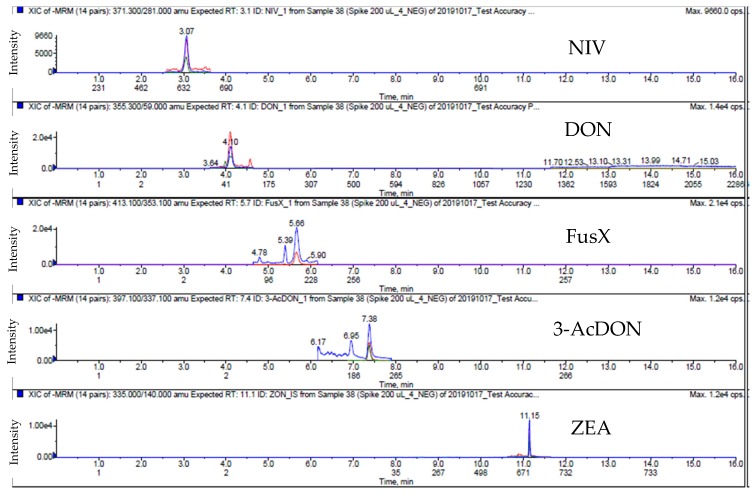
Extract ion chromatogram (EIC) of spiked swine feed samples in ESI (−); 10 ng/g for ZEA, 400 ng/g for DON, 3-AcDON, NIV and FuSX.

**Figure 5:**
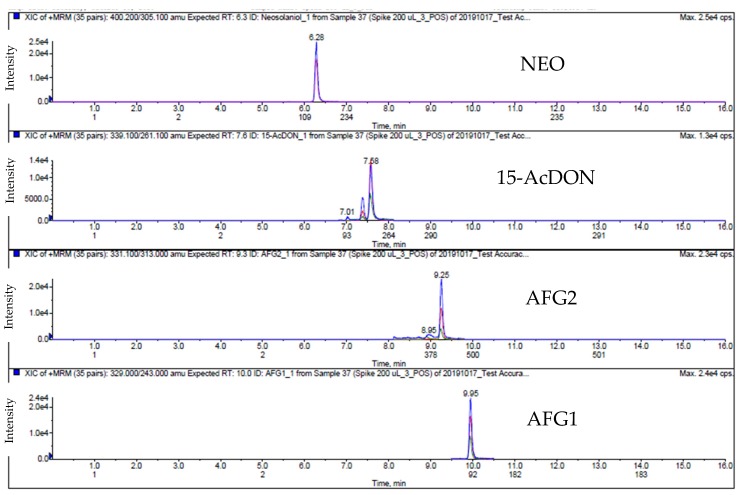

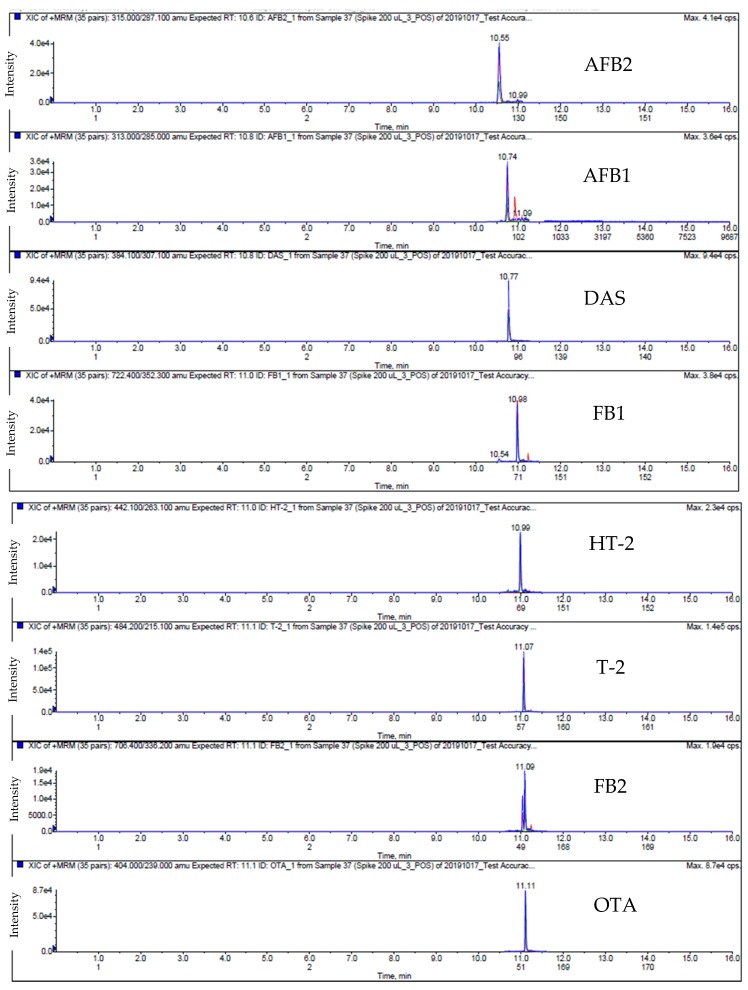
Extract Ion Chromatogram (EIC) of spiked swine feed samples in ESI (+); 10 ng/g for AFB1, AFB2, AFG1 and AFG2, with 80 ng/g for T-2, HT-2, DAS, NEO, and OTA, 400 ng/g for 15-AcDON, 300 ng/g for FB1 and 90 ng/g for FB2.

The optimized QuEChERS procedure was performed with slight modifications from Dzuman et al. (2014) [8]. Three different percentages of FA, consisting of 0.5%, 1.0% and 2.0% at soaking step were evaluated for use in the sample extraction. In addition, 1.0% FA showed the best extraction efficiency and provided satisfied recovery values that were better than those for 0.5% and 2.0% FA for all analytes (Figure 3). In short, the test sample of 1.0 g and soaking solvent of 1% FA were added with stable isotope dilution (internal standard) after the sample had been cleaned up using dSPE. The performance of the optimized QuEChERS method was in line with previous publications [8,22]. The results for spiked swine, poultry and dairy feeds are shown in Figure 4 and Figure 5, respectively.

### 2.3. Method Validation

The results of linearity and sensitivity are reported in Table S1. The method produced good linearity over the relevant working range, with the *r*^2^ value being greater than 0.995. The limit of detection (LOD) values in the matrices ranged from 0.25 ng/g for the aflatoxins in poultry feed matrices to 40.0 ng/g for DON in dairy feed matrices, respectively. The limit of quantification (LOQ) ranged from 0.5 ng/g for aflatoxins in poultry feed matrices to 100.0 ng/g for DON in dairy and poultry feed matrices (Table S1). The LOQ parameters showed the lower amount for some of trichothecenes mycotoxin such as 15-AcDON (50 ng/g), NIV (40 ng/g), FusX (40 ng/g), HT-2 [8 ng/g] and NEO (8 ng/g) in dairy feed matrix with the previous report [8] and for aflatoxin group; AFB1 (80 ng/g) AFB2, AFG1 and AFG2 (4 ng/g) [9]. The recovery and precision values were with the acceptable criteria, in the range 70%–125% and the %RSD values were less than 20% [32] for all 17 mycotoxins, as summarized in Tables S2–S4 for the swine, poultry and dairy feeds, respectively. The identification requirement of the relative ion ratio from sample extracts was lower than 30% for all 17 mycotoxins [33].

### 2.4. Matrix Effect Study

**Figure 6:**
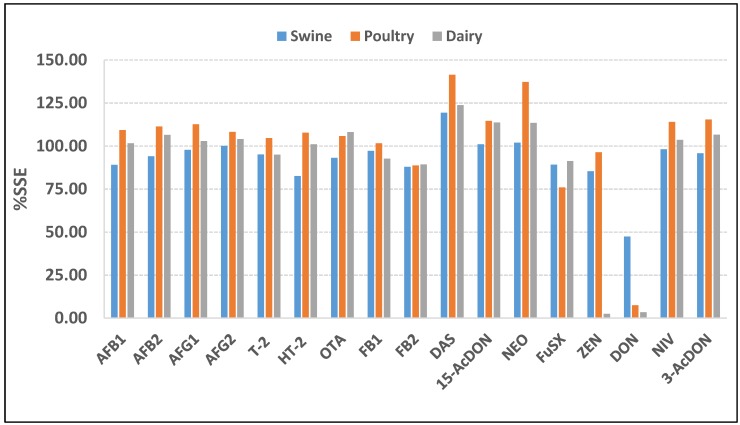
Signal suppression-enhancement (%SSE) for mycotoxin comparison between matrix-matched and solvent calibration using isotopically internal standard (ISTD) for three sample matrices.

The study used %SSE to evaluate the matrix effects in the three types of feed matrices. If the suppression or enhancement was marginal, the %SSE would be very close to 100%; if there was strong suppression/enhancement, the %SSE would deviate from 100%. In the swine feed samples, the %SSE was in the range 82.5%–119.3%, except for DON which exhibited strong signal suppression with the %SSE less than 50% (47.4%). In the poultry feed samples, the %SSE was in the range 76%–115.4%, except for DON which exhibited strong signal suppression (%SSE 7.49%), with strong signal enhancement for NEO and DAS (%SSE 137% and 142%, respectively). In the dairy feed samples, the %SSE was in the range 89.3%–113.7%, except for DON and ZEA which produced the same results as for the poultry feed samples, namely strong signal suppression with %SSE 2.5% and 3.5%, respectively. Regarding signal enhancement, the %SSE was greater than 120% (123.8%) for DAS. The %SSE values of the three types of feed matrices are summarized in Figure 6**.** All results of the matrix effect, the quantification of mycotoxin using matrix matched-calibration as isotopically labeled as the internal standard are necessary.

### 2.5. Occurrence of the Mycotoxins in Animal Feed

**Figure 7:**
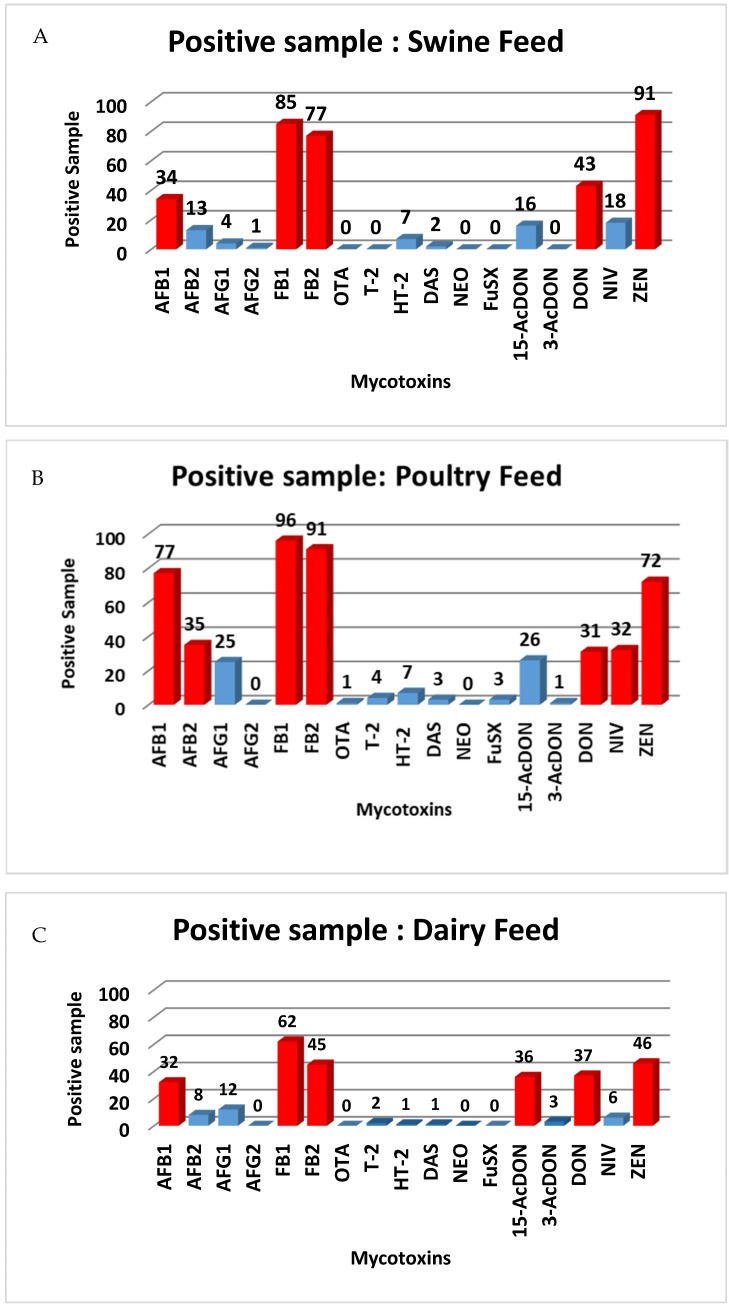
Mycotoxin contamination found in real animal feed samples; **A:** swine feed, **B:** poultry feed and **C:** dairy feed. (Red = >30% contaminated with mycotoxins, Blue = <30% contaminated with mycotoxins)

The developed method was applied to investigate the occurrence of the 17 toxins in 300 feed samples consisting of swine (*n* = 100), poultry (*n* = 100) and dairy feeds (*n* = 100). In the swine feed samples, more than 75% were contaminated with *Fusarium* mycotoxin, especially FB2 (77%), (FB1 85%) and ZEA (91%). The other mycotoxins contaminating the feed samples were: DON (43%), AFB1 (34%), NIV (18%), 15-AcDON (16%), AFB2 (13%), HT-2 (7%), AFG1 (4%), DAS (2%) and AFG2 (1%). However, T-2, NEO, FusX and OTA were not detectable in the swine feed samples. The results were consistent with the contaminants prevalent for *Fusarium* mycotoxin in a previous report [26,34]. In the poultry feed samples, there was a somewhat similar situation for AFB1 (77%) and ZEA (72%) in line with a previous publication [1] with a range of 0.27%–326.4 μg/kg for AFB1 and 1.3–235.8 μg/kg for ZEA, respectively. The FBs were the most contaminants in the poultry samples (90%) ranges of 16.0–2645.5 μg/kg for FB1 and 6.4–573.3 μg/kg for FB2. The trichothecene mycotoxins, especially for type B, consisting of DON, NIV and 15-AcDON were found in more than 25% of samples, but type A trichothecene, consisting of T-2 HT-2 and DAS were found at a lower level (7%) for positive samples. The dairy feed samples had the same prevalent contaminants as the swine feed samples but with lower concentrations of the *Fusarium* mycotoxins, especially for FB2 (45%), (FB1 62%) and ZEA (46%). However, AFG2, NEO, FusX and OTA were not detectable in the dairy feed samples. (Table 1 and Figure 7). The mycotoxin levels in the feed samples almost complied with the EU regulation with exceptions in the poultry and dairy feed samples. Specifically, in the poultry feed samples, the contamination of AFB1 was higher than the regulatory limit (20 ng/g) in three samples (range 24.4–32.64 ng/g), while contamination with AFB1 was higher than 5 ng/g for four samples (range 6.58–14.88 ng/g). For the extracted ion chromatogram (EIC) of some naturally contaminated samples were shown in Figures S1 and S2, respectively. 

## 3. Conclusions

Our results demonstrated the successful development of a stable isotope dilution based on a QuEChERS sample preparation protocol and the LC-ESI-MS/MS method for the simultaneous determination of 17 mycotoxins in animal feed samples. This method was an excellent tool for the unambiguous identification of the 17 selected mycotoxins in the sampled swine, poultry and dairy feeds. The developed method was successfully validated according to the SANTE/11813/2017 standard and was applied to real feed samples. The results showed contamination by multiple mycotoxins with *Fusarium* mycotoxins such as FBs, ZEA, type B trichothecene (especially DON) and *Aspergillus* mycotoxins (especially AFB1) co-occurred most commonly in the animal feeds. The mycotoxin levels in feed samples almost complied with the EU regulations. However, further studies with a larger sample size are needed to confirm these data.

## 4. Materials and Methods

### 4.1. Reagents and Materials

The LC-MS/MS grade reagents, consisting of ammonium acetate, ammonium formate, formic acid, acetic acid, methanol (MeOH) and acetonitrile (ACN) were purchased from Fluka (St.Louis, MO., USA). Anhydrous magnesium sulfate (MgSO_4_) and sodium chloride (NaCl) were purchased from Merck (Darmstadt, Germany). Bondesil C_18_ sorbent for dispersive solid-phase extraction clean-up was purchased from Agilent Technologies (Santa Clara, CA, USA). Deionized water was produced using a Milli-Q system (Millipore; Bedford. MA. USA).

### 4.2. Unlabeled Analytical Standards

There were 17 analytical standards of mycotoxin used in the experiments: 1) *Fusarium* toxins: nivalenol, deoxynivalenol, T-2 toxin, HT-2 toxin and zearalenone were obtained from Biopure (Romer Labs, Tulln, Austria) and the other *Fusarium* toxins consisting of 15-acetyldeoxynivalenol, 3-acetyldeoxynivalenol, fusarenon X, neosolaniol, fumonisin B1 and B2 were obtained from Trilogy Lab (Washington, MO., USA) and diacetoxyscirpenol from Cayman Chemical (Cayman Chemical Ltd., Michigan, MI, USA); 2) *Aspergillus* and *Penicillium* toxins: aflatoxins (B1, B2, G1, G2) were obtained from Sigma Aldrich (St. Louis, MO, USA) and ochratoxin A from Biopure (Romer Labs, Tulln, Austria).

### 4.3. Isotopically Internal Standards

There were 15 stable isotope labeled internal standards: [^13^C_17_]-aflatoxin B1 (500 ng/mL), [^13^C_17_]-aflatoxin B2 (500 ng/mL), [^13^C_17_]-aflatoxin G1 (500 ng/mL), [^13^C_17_]-aflatoxin G2 (500 ng/mL), [^13^C_15_]-deoxynivalenol (25,000 ng/mL), [^13^C_34_]-fumonisin B1 (25,000 ng/mL), [^13^C_34_]-fumonisin B2 (10,000 ng/mL), [^13^C_20_]-ochratoxin A (10,000 ng/mL), [^13^C_24_]-T-2 toxin (25,000 ng/mL), [^13^C_22_]-HT-2 toxin (25,000 ng/mL), [^13^C_18_]-zearalenone (25,000 ng/mL), [^13^C_17_]-3-acetyl-deoxynivalenol (25,000 ng/mL), [^13^C_17_]-15-acetyl-deoxynivalenol (10,000 ng/mL), [^13^C_19_]-diacetoxyscirpenol (25,000 ng/mL) and [^13^C_15_]-nivalenol (25,000 ng/mL) were purchased from Biopure (Romer Labs, Tulln, Austria).

### 4.4. Preparation of Standards Solution

Combinations of the unlabeled standard mycotoxin stock solutions were prepared in methanol to provide a working standard solution at different concentrations: 2000 ng/mL for DON, 15-AcDON, 3-AcDON, NIV and FusX; 400 ng/mL for T-2, HT-2, DAS, NEO and OTA; 50 ng/mL for AFB1, AFB2, AFG1, AFG2 and ZEA; 1500 ng/mL for FB1; and 450 ng/mL for FB2. For the purpose of method validation for spiking experiments, working standard solutions were immediately prepared and stored in amber vials at −20 °C for one week.

### 4.5. Preparation of Isotopically Internal Standards

The concentration of isotopically internal standard (ISTD) working solutions were: 10.0 ng/mL for [^13^C_17_]-AFB1, [^13^C_17_]-AFB2, [^13^C_17_]-AFG1 and [^13^C_17_]-AFG2; 50.0 ng/mL for [^13^C_34_]-FB2 and [^13^C_18_]-ZEA; 125.0 ng/mL for [^13^C_24_]-T-2 toxin and [^13^C_19_]-DAS; 200.0 ng/mL for [^13^C_17_]-15-AcDON and [^13^C_20_]-OTA; 250.0 ng/mL for [^13^C_34_]-FB1 and [^13^C_22_]-HT-2; and 500 ng/mL for [^13^C_15_]-DON, [^13^C_15_]-NIV and [13C17]-3-AcDON.

### 4.6. Feed Samples

The 300 feed samples consisting of swine feed (*n* = 100), poultry feed (*n* = 100) and dairy feed (*n* = 100) were randomly collected from animal farms in different regions of Thailand. All samples were ground with rotor milling ZM200 (Retsh GmbH, Hann, Germany) into fine powder (0.50 mm) and stored at −20 °C before analysis.

### 4.7. QuEChERS-Based Procedure

The sample preparation protocol applying the QuEChERS-base procedure was developed based on Dzuman et al. [8] Briefly, 1 g of homogenized feed sample was weighed into a 50-mL polypropylene (PP) centrifugation tube, followed by the addition of 10 mL of 1% aqueous formic acid solution. The tube was closed and the sample was allowed to soak for 30 min. Then, 10 mL of acetonitrile was added into the soaked sample and shaken using a laboratory shaker (IKA Labortechnik; Staufen, Germany) for 30 min at 240 RPM. The phase partition was induced by the addition of 1 g NaCl and 4 g of MgSO_4_. The tube was immediately shaken for 30 s to prevent coagulation of the MgSO_4_ and then centrifuged (Kubota; Tokyo, Japan) for 5 min at 10,000 RPM. A sample of 2 mL of the acetronitrile phase was placed into a 15 mL PP tube containing 0.1 g of C_18_ silica sorbent and 0.3 g of MgSO_4_ which were mixed and then centrifuged for 1 min. The purified extract was evaporated to dryness at 40 °C. The residue was reconstituted in 960 μL 20% MeOH and then 40 μL of [^13^C]-ISTD working solution were added. The mixture was passed through a 0.22 μm nylon filter before being used in the LC-MS/MS analysis.

### 4.8. LC-MS/MS Analysis

The 17 target mycotoxins were analyzed using the UHPLC-MS/MS method. Chromatographic separation was developed according to [22]. The analysis used an ExionLC™ AD system (AB SCIEX; Toronto, ON, Canada) was equipped with an Accucore analytical column (100 × 2.1 mm i.d., 2.6 μm particle size; Thermo Scientific; San Jose, CA, USA) maintained at 25 °C. The mobile phase differed for the ESI (+) and ESI (−) analyses, with 5 mM ammonium formate and 0.1% formic acid (v/v) both in deionized water (A) and MeOH (B) being used in ESI (+), whereas 5 mM ammonium acetate and 0.1% acetic acid (v/v) both in deionized water (C) and MeOH (D) were used for ESI (−). The gradient elution was identical in both ESI (+) and (−), starting at 0% B/D followed by a linear change to 20% B/D in 4 min and subsequently using linear changes to 40% B/D in 5.5 min and 100% B/D in 10.5 min. Then, the column was washed for 2.5 min with 100% B/D followed by a reconditioning for 3 min using the initial composition of mobile phases. The flow rate was stable at 0.4 mL/min throughout the run and 3 μL of sample extract was injected into the LC-MS/MS system.

The ExionLC™ AD system was coupled to a QTRAP 5500 tandem mass spectrometer (ABSCIEX; Toronto, ON, Canada), equipped with an electrospray (ESI) ion source operated in both positive and negative mode. The ESI (+) ion source parameters were: needle voltage 4500 V; curtain gas 30 psi; nebulizer (Gas1) and turbo gas (Gas2) 55 psi; and turbo gas temperature 500 °C. The ESI (−) source parameters were: needle voltage −4500 V; curtain gas 30 psi; nebulizer (Gas 1) and turbo gas (Gas2) 55 psi; and turbo gas temperature 500 °C. The declustering potential (DP), collision energy (CE) and collision cell exit potential (CXP) were optimized during infusion of individual analytes (10–200 ng/mL) using manual infusion. The MRM transitions of unlabeled mycotoxins and isotopically internal-standard-dependent parameters are summarized in Table 2.

### 4.9. Method Validation

The method performance characteristic parameters (linearity and ranges, accuracy, precision, LOD and LOQ) were determined for the samples of swine, poultry and dairy feeds. The analytes were quantified using an internal matrix-matched calibration standard with post spiking calibration curve for 15 mycotoxins excluding FusX and NEO (using external matrix-matched calibration standard) at the following levels: 1.0–40.0 ng/g (corresponding to 0.1–4.0 ng/mL) for AFB1, AFB2, AFG1, AFG2 and ZEA; 40–1600 ng/g (corresponding to 4–160 ng/mL) for DON, 15-AcDON, 3-AcDON, NIV and FusX; 8–320 ng/g (corresponding to 0.8–32 ng/mL) for T-2, HT-2, DAS, NEO and OTA; 30–1200 ng/g (corresponding to 3–120 ng/mL) for FB1; and 9–360 ng/g (corresponding to 0.9–36 ng/mL) for FB2. The accuracy and precision (repeatability, expressed as relative standard deviation (RSD) in %) were determined within-day by analyzing five replicates at three levels (low, intermediate, high). The inter-day precision was determined at the same level as the within-day precision on three different days. The LOQ values were estimated using the concentration of analytes which provided a signal-to-noise ratio (S/N) greater than 10 and the LOD values were defined as the minimum concentration of analytes which provided S/N values greater than 3. 

### 4.10. Matrix Effect Study

The matrix effects of the QuEChERS-based method were evaluated within three types of feed matrices: swine, poultry and dairy feed. Internal matrix-matched standards were prepared at seven different levels: 1.0–40.0 ng/g (corresponding to 0.1–4.0 ng/mL) for AFB1, AFB2, AFG1, AFG2 and ZEA; 40–1600 ng/g (corresponding to 4–160 ng/mL) for DON, 15-AcDON, 3-AcDON, NIV and FusX; 8–210 ng/g (corresponding to 0.8–32 ng/mL) for T-2, HT-2, DAS, NEO and OTA; 30–1200 ng/g (corresponding to 3–120 ng/mL) for FB1; and 9–360 ng/g (corresponding to 0.9–36 ng/mL) for FB2 by the addition of standard solution into the sample extract. The matrix effects expressing the matrix induced signal suppression/enhancement (SSE%) were defined as percentage ratios of the matrix-matched calibration slope to the solvent calibration slope.

## Figures and Tables

**Table 1:** Occurrence of 17 mycotoxins in swine feed, poultry feed and dairy feed.

Mycotoxin	Swine Feed (*n* = 100)			Poultry Feed (*n* = 100)			Dairy Feed (*n* = 100)		
	Positive Sample	Range (ng/g)	Mean (ng/g)	Positive Sample	Range (ng/g)	Mean (ng/g)	Positive Sample	Range (ng/g)	Mean (ng/g)
AFB1	34	0.52–14.2	1.7	77	0.27–326.4	8.2	32	0.54–14.9	1.6
AFB2	13	0.51–4.1	0.9	35	0.26–49.9	2.4	8	1.5–2.4	0.9
AFG1	4	0.51–1.6	0.5	25	0.25–0.86	0.6	12	0.5–1.6	0.5
AFG2	1	0.66	0.66	ND	ND	ND	ND	ND	ND
T-2	ND	ND	ND	4	2.1–3.2	2.6	2	5.1–5.5	4.5
HT-2	7	6.0–19.3	9.6	7	2.6–10.0	6.6	1	15.23	15.23
OTA	ND	ND	ND	1	3.1	3.1	ND	ND	ND
FB1	85	15.0–464.8	102.4	96	16.0–2645.5	451.7	62	16.2–731.0	88.1
FB2	77	5.0–136.1	31.08	91	6.4–573.3	123.2	45	5.4–252.2	26.1
DAS	2	4.2–5.1	4.7	3	2.8–3.6	3.1	1	4.36	4.36
15-AcDON	16	23.0–83.2	30.8	26	12.8–57.1	31.7	36	20.9–68.2	35.9
NEO	ND	ND	ND	ND	ND	ND	ND	ND	ND
FuSX	ND	ND	ND	3	39.3–61.9	48.0	ND	ND	ND
ZEA	91	0.53–169.2	17.4	72	1.3–235.8	30.2	46	0.73–98.4	26.1
DON	43	20.1–631.9	215	31	28.7–1430.8	304.6	37	50.2–538.8	167.8
NIV	18	21.9–165.4	46.0	32	25.6–626.0	103.4	6	24.0–117.5	51.2
3-AcDON	ND	ND	ND	1	45.6	45.6	3	29.9–46.6	36.0

**Table 2:** MS/MS parameters for determination of 17 mycotoxins and 15 isotope labeled internal standards.

Compounds	Q1 (m/z)	Q3 (m/z)	RT (min)	DP (V)	EP (V)	CE (V)	CXP (V)
AFB1	313	285.00 *	10.75	136.0	6.20	33.0	20.0
	313	241		136.0	6.20	51.0	20.0
[^13^C_17_]-AFB1	330.1	255.1	10.75	94.5	7.65	50.3	13.5
AFB2	315	287.10 *	10.59	141.0	5.07	37.0	20.0
	315	259		141.0	5.07	41.0	24.0
[^13^C_17_]-AFB2	332.1	303.2	10.59	97.1	7.59	36.8	25.0
AFG1	329	243.00 *	9.99	138.0	7.77	34.0	20.0
	329	200		138.0	7.77	51.0	18.0
[^13^C_17_]-AFG1	346.2	212.1	9.99	99.9	4.90	54.2	18.1
AFG2	331.1	313.00 *	9.29	136.0	3.87	35.0	18.0
	331.1	189		136.0	3.87	57.0	12.0
[^13^C_17_]-AFG2	348.1	259.2	9.29	95.9	5.49	43.6	16.1
T-2	484.2	215.10 *	11.08	69.9	6.05	25.0	14.8
	484.2	185.1		69.9	6.05	28.9	16.1
[^13^C_24_]-T-2	508.3	229.2	11.08	77.6	7.77	26.7	16.8
HT-2	442.1	263.10 *	11.00	66.5	4.86	18.0	24.0
	442.1	215		66.5	4.86	18.6	24.5
[^13^C_22_]-HT-2	464.3	229.1	11.00	70.0	6.10	18.1	17.5
OTA	404	239.00 *	11.12	80.0	5.78	30.9	21.0
	404	221		80.0	5.78	47.4	17.0
[^13^C_20_]-OTA	424.2	250.1	11.12	85.1	7.95	32.3	21.4
FB1	722.4	352.30 *	10.99	165.0	7.85	49.0	26.0
	722.4	334.3		165.0	7.85	55.0	20.0
[^13^C_34_]-FB1	756.5	356.4	10.99	83.7	7.73	55.9	17.5
FB2	706.4	336.20 *	11.12	165.0	8.00	53.0	16.0
	706.4	318.3		165.0	8.00	53.0	18.0
[^13^C_34_]-FB2	740.6	358.4	11.12	87.3	11.00	50.7	23.2
DAS	384.1	307.10 *	10.78	68.8	7.67	15.60	22.70
	384.1	247.2		68.8	7.67	18.60	17.90
[^13^C_19_]-DAS	403.2	324.1	10.78	70.2	5.67	15.60	10.10
15-AcDON	339.1	261.10 *	7.61	68.8	6.16	14.20	12.90
	339.1	137		68.8	6.16	15.80	19.90
[^13^C_17_]-15-AcDON	373.2	338.1	7.61	60.9	8.27	17.60	23.30
Neosolaniol	400.2	305.10 *	6.3	64.2	5.94	16.40	9.40
	400.2	215.1		64.2	5.94	23.80	14.90
DON	355.3	59.00 *	4.13	−58.7	−4.79	−20.37	−17.09
	355.3	295		−58.7	−4.79	−13.84	−8.34
[^13^C_15_]-DON	370.4	310.1	4.13	−57.7	−2.72	−14.37	−9.14
NIV	371.3	281.00 *	3.11	−61.7	−2.97	−20.26	−24.79
	371.3	311		−61.7	−2.97	−14.29	−20.01
[^13^C_15_]-NIV	386	295.2	3.11	−50.2	−3.76	−20.60	−8.37
3-AcDON	397.1	337.10 *	7.38	−60.1	−5.91	−20.22	−9.04
	397.1	307		−60.1	−5.91	−12.47	−9.94
[^13^C_17_]-3-AcDON	414	323.2	7.38	−59.3	−5.60	−20.04	−9.25
ZEA	317	175.00 *	11.14	−172.9	−3.57	−32.59	−9.20
	317	131		−172.9	−3.57	−38.00	−13.00
[^13^C_18_]-ZEA	335	140	11.14	−161.9	−8.80	−39.16	−7.28
FusX	413.1	353.10 *	5.65	−50.0	−10.00	−14.00	−17.00
	413.1	263	5.65	−50.0	−10.00	−20.00	−17.00

Note: * = Quantifier.

## Supplementary Materials

The following are available online at https://www.mdpi.com/2072-6651/12/4/253/s1, Figure S1: Extract Ion Chromatogram (EIC) of naturally contaminated of AFB1 at 326.4 ng/g in poultry feed sample; **A**: Quantifier, **B**: Qualifier and **C**: IS-AFB1. Figure S2. EIC of naturally contaminated of AFB1 at 14.88 ng/g in dairy feed sample, Table S1. Linearity ranges, limit of detection (LOD) and limit of quantification (LOQ) of the optimized UHPLC-MS/MS method for simultaneous determination of mycotoxins, Table S2. Accuracy and precision for mycotoxin determination in optimal LC-MS/MS conditions for swine feed samples, Table S3. Accuracy and precision for mycotoxin determination in optimal LC-MS/MS conditions for poultry feed samples, Table S4. Accuracy and precision for mycotoxin determination in optimal LC-MS/MS conditions for dairy feed samples.
