# Supplementary material for: Simultaneous Determination of Multiple Mycotoxins in Swine, Poultry and Dairy Feeds Using Ultra High Performance Liquid Chromatography-Tandem Mass Spectrometry
Source: Toxins (Basel). 2020 Apr 13;12(4):253. doi: 10.3390/toxins12040253 (PMC7232461; doi:10.3390/toxins12040253)
Supplement: Supplementary file 1 [file toxins-12-00253-s001.pdf]

Supplementary Materials: Simultaneous Determination of Multiple Mycotoxins in Swine, Poultry and Dairy Feeds Using Ultra High Performance Liquid Chromatography-Tandem Mass Spectrometry

Kraiwut Nualkaw, Saranya Poapolathep, Zhaowei Zhang, Qi Zhang, Mario Giorgi, Peiwu Li, Antonio Francesco Logrieco, and Amnart Poapolathep *

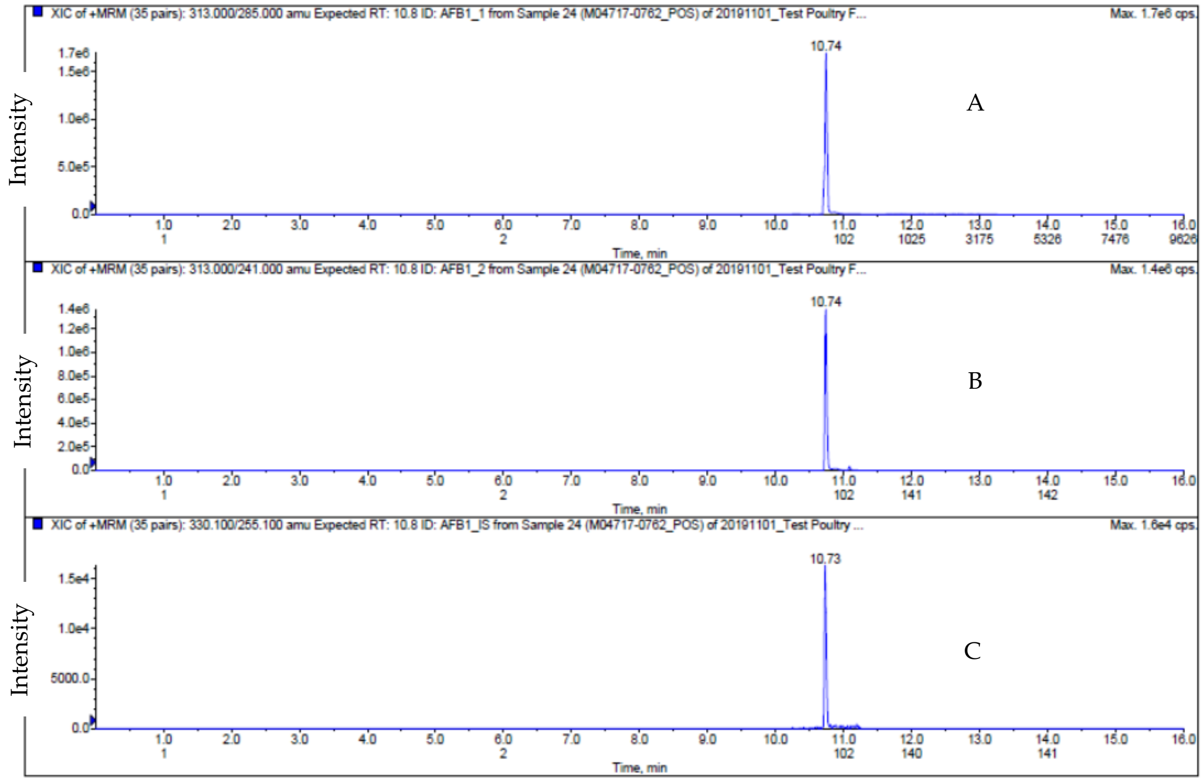

Figure S1. Extract Ion Chromatogram (EIC) of naturally contaminated of AFB1 at 326.4 ng/g in poultry feed sample; **A**: Quantifier, **B**: Qualifier and **C**: IS-AFB1.

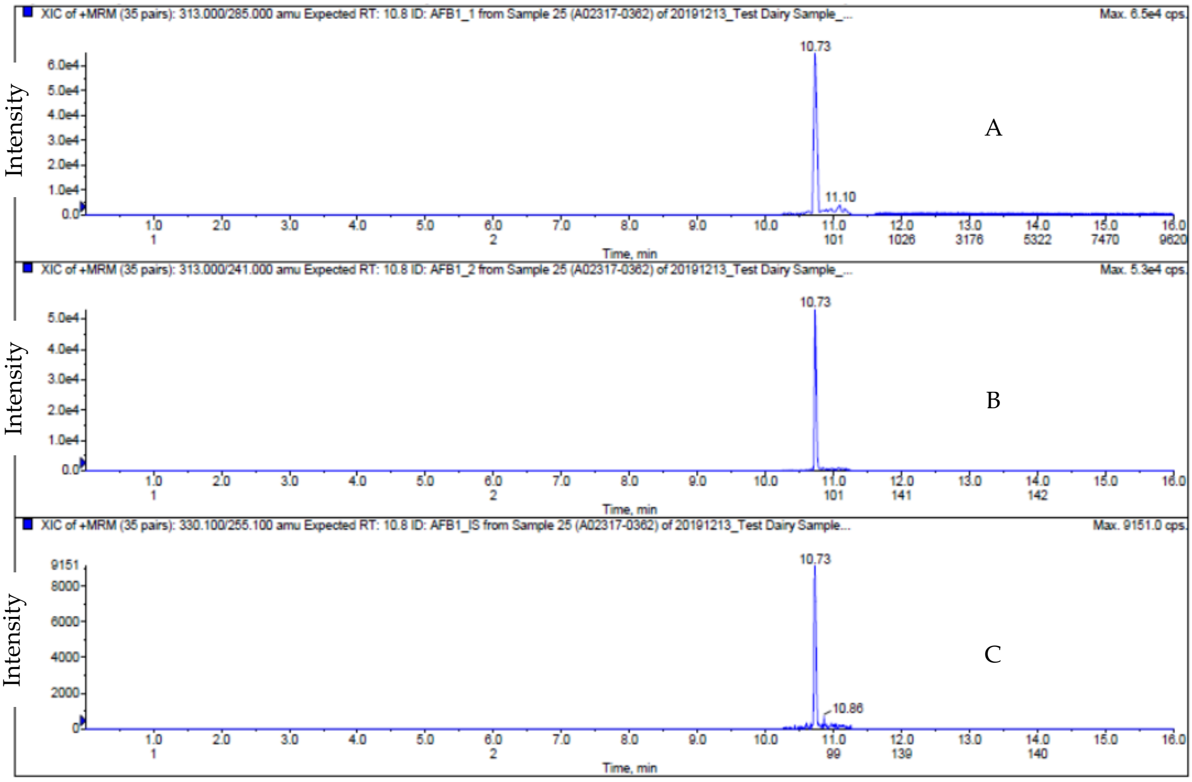

Figure S2. Extract Ion Chromatogram (EIC) of naturally contaminated of AFB1 at 14.88 ng/g in dairy feed sample. **A**: Quantifier, **B**: Qualifier and **C**: IS-AFB1.

Table S1. Linearity ranges, limit of detection (LOD) and limit of quantification (LOQ) of the optimized UHPLC-MS/MS method for simultaneous determination of mycotoxins.

Mycotoxin	Limit of Detection (LOD), ng/g			Limit of Quantification (LOQ), ng/g			Calibration Range, ng/g		
	Swine Feed	Poultry Feed	Dairy Feed	Swine Feed	Poultry Feed	Dairy Feed	Swine Feed	Poultry Feed	Dairy Feed
AFB1	0.5	0.25	0.5	1.0	0.5	1.0	1.0-40.0	0.5-40.0	1.0-40.0
AFB2	0.5	0.25	0.5	1.0	0.5	1.0	1.0-40.0	0.5-40.0	1.0-40.0
AFG1	0.5	0.25	0.5	1.0	0.5	1.0	1.0-40.0	0.5-40.0	1.0-40.0
AFG2	0.3	0.25	0.5	0.5	0.5	1.0	0.5-40.0	0.5-40.0	1.0-40.0
15-AcDON	20.0	10.0	20.0	40.0	20.0	40.0	40.0-1600	20.0-1600	40.0-1600
T-2	4.0	2.0	4.0	8.0	4.0	8.0	8.0-320	4.0-320	8.0-320
HT-2	4.0	2.0	4.0	8.0	8.0	8.0	8.0-320	8.0-320	8.0-320
DAS	4.0	2.0	4.0	8.0	4.0	8.0	8.0-320	4.0-320	8.0-320
NEO	4.0	2.0	4.0	8.0	4.0	20.0	8.0-320	4.0-320	20.0-320
OTA	4.0	2.0	4.0	8.0	4.0	8.0	8.0-320	4.0-320	8.0-320
FB1	15.0	15.0	15.0	30.0	30.0	30.0	30.0-1200	30.0-1200	30.0-1200
FB2	4.5	4.5	4.5	9.0	9.0	9.0	9.0-360	9.0-360	9.0-360
FuSX	20.0	10.0	20.0	40.0	20.0	40.0	40.0-1600	20.0-1600	40.0-1600
ZEN	0.5	0.25	0.5	1.0	1.0	1.0	1.0-40.0	1.0-40.0	1.0-40.0
DON	20.0	20.0	40.0	40.0	100.0	100.0	40.0-1600	100.0-1600	100.0-1600
NIV	20.0	20.0	20.0	40.0	40.0	40.0	40.0-1600	40.0-1600	40.0-1600
3-AcDON	20.0	20.0	20.0	100.0	40.0	40.0	100-1600	40.0-1600	40.0-1600

Table S2. Accuracy and precision for mycotoxin determination in optimal LC-MS/MS conditions for swine feed samples.

Mycotoxin	Spiked Level, (ng/g)	Swine Feed		
		%Recovery, (%)	Intra-Day Precision, (%RSD)	Inter-Day Precision, (%RSD)
FuSX	200.0	103.0	3.67	10.69
	400.0	101.0	1.46	8.70
	800.0	91.0	1.14	8.64
ZEN	2.5	100.0	12.02	15.53
	5.0	88.0	17.95	17.71
	10.0	83.0	4.73	14.19
DON	200.0	88.0	1.68	11.62
	400.0	91.0	4.36	10.00
	800.0	81.0	1.07	15.34
NIV	200.0	87.0	1.69	2.95
	400.0	91.0	2.96	4.52
	800.0	89.0	4.97	5.18
3-AcDON	200.0	106.0	6.43	8.77
	400.0	107.0	4.22	11.24
	800.0	101.0	2.11	15.25
AFB1	2.5	99.0	10.29	12.13
	5.0	90.0	6.16	4.72
	10.0	93.0	10.00	12.89
AFB2	2.5	96.0	2.33	9.08
	5.0	99.0	4.38	7.78
	10.0	99.0	3.96	7.98
AFG1	2.5	94.0	7.56	6.87
	5.0	89.0	3.40	3.58
	10.0	92.0	6.96	7.64
AFG2	2.5	95.0	1.63	4.35
	5.0	102.0	2.94	7.51
	10.0	104.0	0.52	9.25
15-AcDON	200.0	103.0	4.97	7.89
	400.0	105.0	2.58	12.65
	800.0	93.0	2.54	12.32
T-2	20.0	92.0	5.88	10.79
	40.0	96.0	6.17	9.65
	80.0	101.0	3.16	11.41
HT-2	20.0	104.0	5.35	14.85
	40.0	95.0	6.16	14.64
	80.0	95.0	7.69	17.10
DAS	20.0	99.0	6.35	7.15
	40.0	104.0	2.34	8.51
	80.0	107.0	4.62	11.39
NEO	20.0	96.0	11.53	9.88
	40.0	95.0	4.69	7.13
	80.0	96.0	3.31	9.69
OTA	20.0	76.0	5.85	7.50
	40.0	81.0	11.82	10.41
	80.0	80.0	4.82	13.56
FB1	75.0	82.0	8.58	10.43
	150.0	79.0	6.01	7.13
	300.0	89.0	9.84	11.79
FB2	22.5	83.0	7.35	10.80
	45.0	76.0	3.74	5.24
	90.0	83.0	3.39	8.70

Table S3. Accuracy and precision for mycotoxin determination in optimal LC-MS/MS conditions for poultry feed samples.

Mycotoxin	Spiked Level, (ng/g)	Poultry Feed		
		%Recovery, (%)	Intra-Day Precision, (%RSD)	Inter-Day Precision, (%RSD)
FuSX	100.0	102	3.85	5.47
	200.0	104	6.65	5.95
	400.0	116	0.64	2.83
ZEN	2.5	85	13.18	9.70
	5.0	99	16.11	12.88
	10.0	107	3.42	3.58
DON	100.0	105	9.96	11.64
	200.0	96	12.50	11.20
	400.0	96	5.93	9.94
NIV	100.0	83	3.97	4.93
	200.0	85	5.67	6.44
	400.0	95	3.49	2.77
3-AcDON	100.0	99	6.80	6.34
	200.0	105	7.90	7.72
	400.0	114	1.66	3.30
AFB1	2.5	95	4.74	6.62
	5.0	91	5.03	8.20
	10.0	99	4.77	5.92
AFB2	2.5	100	4.44	3.82
	5.0	105	7.14	6.27
	10.0	116	2.62	2.42
AFG1	2.5	96	1.93	3.46
	5.0	97	6.60	5.83
	10.0	106	2.86	3.20
AFG2	2.5	103	3.38	2.95
	5.0	109	4.89	5.09
	10.0	117	1.37	2.24
15-AcDON	100.0	103	3.69	6.40
	200.0	107	6.60	5.28
	400.0	112	1.29	1.76
T-2	20.0	99	7.90	6.63
	40.0	107	4.39	5.70
	80.0	115	1.80	2.79
HT-2	20.0	96	9.01	10.81
	40.0	107	4.65	5.29
	80.0	114	1.31	4.98
DAS	20.0	104	5.77	6.89
	40.0	106	7.28	6.30
	80.0	117	2.97	2.86
NEO	20.0	92	2.64	8.83
	40.0	96	5.87	13.60
	80.0	102	9.69	9.15
OTA	20.0	95	3.69	5.77
	40.0	102	5.98	6.55
	80.0	112	2.78	3.75
FB1	75.0	82	12.15	9.94
	150.0	75	3.17	6.68
	300.0	79	6.06	8.87
FB2	22.5	88	8.33	8.26
	45.0	87	11.95	9.79
	90.0	101	8.56	7.39

Table S4. Accuracy and precision for mycotoxin determination in optimal LC-MS/MS conditions for dairy feed samples.

Mycotoxin	Spike Level, (ng/g)	Dairy Feed		
		%Recovery, (%)	Intra-Day Precision, (%RSD)	Inter-Day Precision, (%RSD)
FuSX	100.0	108	1.22	3.46
	200.0	103	1.63	2.26
	400.0	104	2.83	2.92
ZEN	2.5	88	2.91	5.15
	5.0	82	6.09	6.07
	10.0	78	9.00	7.23
DON	100.0	99	12.82	11.33
	200.0	94	9.13	7.80
	400.0	105	4.86	6.87
NIV	100.0	89	2.05	5.54
	200.0	82	2.30	3.15
	400.0	86	5.22	4.68
3-AcDON	100.0	109	3.07	4.19
	200.0	102	1.69	3.21
	400.0	98	1.50	2.68
AFB1	2.5	97	1.18	4.85
	5.0	89	2.86	4.11
	10.0	84	4.47	4.29
AFB2	2.5	104	4.09	3.01
	5.0	101	1.67	2.70
	10.0	98	4.04	4.01
AFG1	2.5	105	1.47	2.62
	5.0	96	4.16	4.11
	10.0	90	3.17	3.81
AFG2	2.5	106	0.29	2.70
	5.0	102	2.13	3.53
	10.0	98	2.65	3.58
15-AcDON	100.0	104	5.71	5.89
	200.0	102	3.42	5.79
	400.0	96	3.31	5.59
T-2	20.0	102	1.13	3.15
	40.0	95	6.12	5.39
	80.0	99	8.56	6.15
HT-2	20.0	105	4.94	4.76
	40.0	100	7.99	7.53
	80.0	96	5.43	13.01
DAS	20.0	108	5.17	4.26
	40.0	99	1.96	2.49
	80.0	93	3.76	5.62
NEO	20.0	108	4.17	5.08
	40.0	103	4.32	3.52
	80.0	113	11.69	9.71
OTA	20.0	90	1.93	5.07
	40.0	80	5.79	6.74
	80.0	76	7.41	6.10
FB1	75.0	90	2.53	3.82
	150.0	78	7.20	6.17
	300.0	76	6.33	5.60
FB2	22.5	90	3.50	8.03
	45.0	88	7.92	10.21
	90.0	84	8.25	10.36
